# Highly Selective, Aptamer-Based, Ultrasensitive Nanogold Colorimetric Smartphone Readout for Detection of Cd(II)

**DOI:** 10.3390/molecules24152745

**Published:** 2019-07-29

**Authors:** Lu Xu, Jun Liang, Yonghui Wang, Shuyue Ren, Jin Wu, Huanying Zhou, Zhixian Gao

**Affiliations:** 1 Tianjin Key Laboratory of Risk Assessment and Control Technology for Environment and Food Safety, Tianjin Institute of Environmental & Operational Medicine, Tianjin 300050, China; 2 State Key Laboratory of Food Nutrition and Safety, Tianjin University of Science and Technology, Tianjin 300457, China

**Keywords:** aptamer, nanogold, colorimetry, smartphone readout, Cd(II)

## Abstract

A highly selective and sensitive method for Cd(II) detection was developed based on aptamer and gold nanoparticles (AuNPs) combined with a colorimetric smartphone readout. The experimental conditions such as reaction time of polydiene dimethyl ammonium chloride (PDDA) and AuNPs, PDDA dose, time of aptamer and PDDA incubation, and aptamer concentration were optimized. Under the optimized conditions, the color and red(R) value of the solution was concentration-dependent on Cd(II). The proposed method exhibited a linear range of 1–400 ng/mL (r^2^ = 0.9794) with a limit of detection (LOD) of 1 ng/mL. This method had been successfully applied to test and quantify Cd(II) in water and rice samples, and the results were in full agreement with those from the atomic absorption spectrometer. Therefore, low-cost colorimetry demonstrated its potential for practical application in visual or quantitative detection with a smartphone. This approach can be readily applied to other analytes.

## 1. Introduction

Nowadays, contamination of heavy metals in waters and agricultural soils has become a severe hazard worldwide [1]. Among many toxic metals, Cd(II) is one of the most hazardous metals with a recognized high toxicity at very low exposure levels. The US Environmental Protection Agency (EPA) has defined a maximum concentration of 5 ng/mL for Cd(II) in drinking water. Intake of Cd(II) at very low concentrations could affect the normal function of enzyme systems in liver and kidney organs and even may cause chronic or acute degradation of human organs [2,3]. Cd(II) invades the environment in different ways [4]. Persistence of Cd(II) leads to bioaccumulation, which shows an important mobility from environmental matrices, such as soils and sediments, where it tends to accumulate (in particular, in seabed sediments close to industrial and urban zones) [5]. Cd(II) in soil, water, air, and logistics transportation can cause food spoilage [6,7]. Thus, rapid identification and quantification of Cd(II) in food and environmental samples has attracted widespread attention and is of paramount importance. The main methods for detecting trace amounts of Cd(II) are graphite furnace atomic absorption spectrometry (GFAAS) [8], inductively coupled plasma mass spectrometry (ICP-MS) [9], electrochemical sensing [10,11,12], surface-enhanced Raman spectroscopy (SERS) [13], fluorescence [14], and colorimetry [15]. Among these methods, GFAAS, ICP-MS, and SERS have excellent sensitivities and wide availabilies in laboratories, but colorimetry is more suitable for on-site screening. A number of azolylazo-based reagents have been prepared and investigated for detecting cadmium using colorimetry. These reagents are sensitive and can meet trace cadmium analysis requirements, but the reagents have poor selectivity [16]. Specifically, the reagents can react with many transition metals such as Cu, Zn, Cd, Cr, Co, Ni, and Hg with high sensitivity under similar conditions.

Currently, noble metal nanomaterials have attracted great interest due to their excellent physical and chemical properties [17]. Of all kinds of noble metal nanomaterials, gold nanoparticles (AuNPs) have shown great potential for use in different applications, such as immunochromatography [18,19] and in other sensors [20,21], due to their high color correspondence and controllable shape. Although methods based on AuNPs have extensive applicability, it is still not specific enough. The application of AuNPs as a signal element relies on the use of biorecognition elements, such as antibodies, oligonucleotides, enzymes, etc., which can transduce an AuNPs signal change in the presence of the molecule of interest. Aptamers are short DNA or RNA chains, which selectively bind to a target molecule with high affinity. Compared with antibodies and enzymes, aptamers are resistant to heat, pH variation, and chemically harsh conditions, which makes them more suitable for practical applications [22].

**Figure 1:**
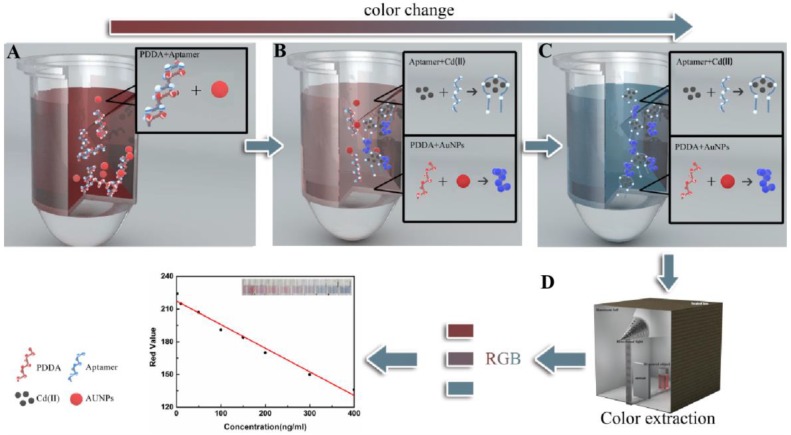
Schematic illustration of the colorimetric method. (**A**) Gold nanoparticles (AuNPs) incubated with aptamer and polydiene dimethyl ammonium chloride (PDDA); the color of the free AuNP solution was wine red; (**B**) AuNPs incubated with the aptamer, PDDA, and few Cd(II); the color of partial aggregation of the AuNP solution changed to reddish; (**C**) AuNPs incubated with the aptamer, PDDA, and sufficient Cd(II); the color of aggregation of the AuNP solution changed to blue; and (**D**) The red(R) values of the solution recorded with a smart phone.

Here, we successfully developed a simple method to measure Cd(II) based on aptamer and AuNPs combined with a colorimetric smartphone readout (Figure 1). The method utilizes an aptamer competitive binding assay of the cationic polymer polydiene dimethyl ammonium chloride solution (PDDA) and Cd(II). AuNPs, as a signal element, rely on free PDDA. The color of the AuNP solution changes from to blue with an increase in Cd(II) concentration. More interestingly, the AuNP solution, which contains signal information, can be photographed by a smartphone equipped with a ColorAssist app. The red(R) values (in the red, green, and blue (RGB) color model) in the image were observed in response to different concentrations of Cd(II). These values were considered as a signal intensity for plotting the standard curve. As can be seen from Figure 1A, the aptamer and PDDA in the solution were combined by electrostatic interaction when there was no target Cd(II). AuNPs were free particles, and the solution was wine red; Figure 1B had shown that the target Cd(II) was added but insufficient. Some aptamers could bind to Cd(II), and the remaining aptamers could bind to PDDA, which may lead to the aggregate of excess PDDA and AuNPs. The transmitted light intensity of the solution decreased and blue-shifted in the maximum absorption wavelength. The color of the solution changed to reddish. When sufficient target Cd(II) was added (Figure 1C), the color of solution turned to blue because the aptamer had bound to Cd(II), and free PDDA caused aggregation of AuNPs. In Figure 1D, the R values of the solution were recorded with a smartphone, and the concentrations of Cd(II) was the response. Therefore, the proposed approach may be used for the quantitative detection of Cd(II). This shows the potential of this method for practical application for in situ visualization or quantitative detection of Cd(II) with any smartphone. 

## 2. Results

### 2.1. Characterization of Gold Nanoparticles (AuNPs) with Different Reagents

**Figure 2:**
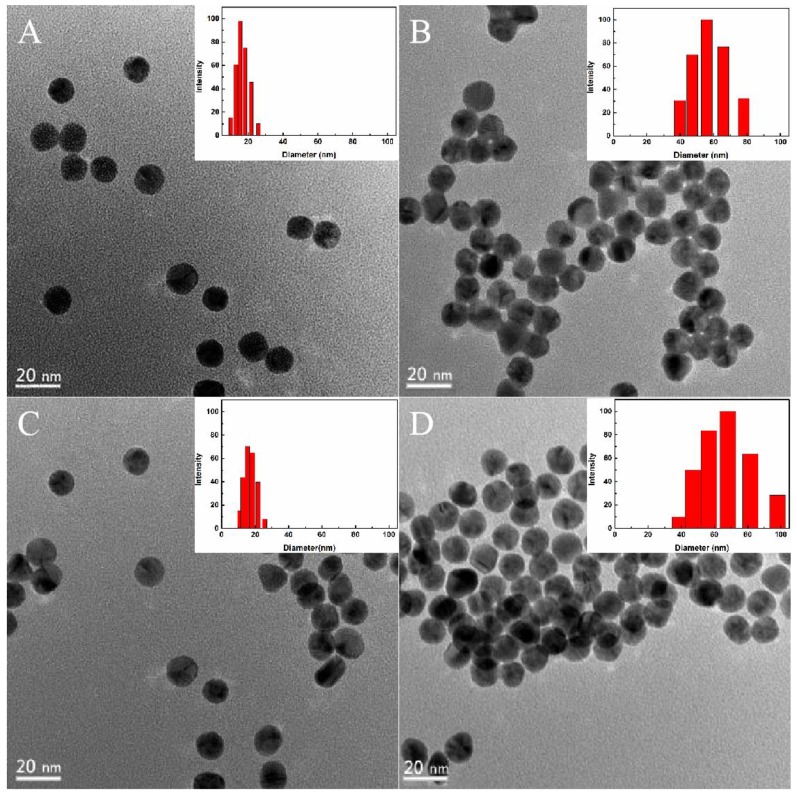
TEM images and particle size distribution of AuNPs added to different materials. (**A**) AuNPs, (**B**) added PDDA, (**C**) added aptamer, and (**D**) added Cd(II).

To verify that PDDA could cause aggregation of AuNPs to achieve indirect determination of Cd(II), a transmission electron microscope (TEM) and laser light-scattering instrument were used to show the microscopic morphology of AuNPs under different conditions (Figure 2). It can be seen that AuNPs had a particle size of approximately 13 nm, a uniform distribution of dimensions, and exhibited a good dispersion state (Figure 2A). AuNPs and PDDA can be combined by electrostatic interaction, and AuNPs were added to PDDA solution, which exhibited a clear aggregation state (Figure 2B). After the addition of aptamer, PDDA and the aptamer formed an aptamer–PDDA complex by electrostatic interaction, and AuNPs exhibited a distinct dispersion state (Figure 2C). The color of the AuNP solution was wine red (Figure 2D) after the addition of Cd(II). At the same time, the particle size of the AuNPs in different states was also analyzed (the data here refers to the particle size distribution of most of the particles in this state), and the particle size analysis is shown in Figure 2. It can be seen that the particle size of the AuNPs in solution was approximately 15–20 nm, and the color of the AuNP solution was burgundy. When the particle size of the aggregated AuNPs increased to 50–80 nm, the color of the AuNP solution changed to blue.

### 2.2. Cd(II)-Binding Aptamer

Cadmium holds a large quantity of electrons distributed in the outer shell, and some of them exist as lone electron pairs, so cadmium can bind to the adjacent T or G bases of the selected aptamer candidates through the coordination bonds between Cd(II) and the O or N of the above bases [15]. As shown in Supplementary Figure S1A, the aptamer could form a stem-loop structure with Cd(II). The loop was a coordinate bond formed between Cd(II) and the adjacent short segments of T or G. The main role of the stem was to maintain ring stability through hydrogen bonds between AT or GC base pairs. To further verify the affinity of the interaction between the aptamer and Cd(II), the Circular Dichroism(CD) spectrum of the solution after cadmium bound to the aptamer was detected by the CD measurement technique. As shown in Supplementary Figure S1B, the aptamer had a negative peak at 240–250 nm and a positive peak at 270–290 nm. After adding Cd(II), the positive peak decreased, the negative peak increased, and the peak position changed. This indicated that Cd(II) could combine well with the aptamer. 

### 2.3. Optimum of Reaction Conditions

**Figure 3:**
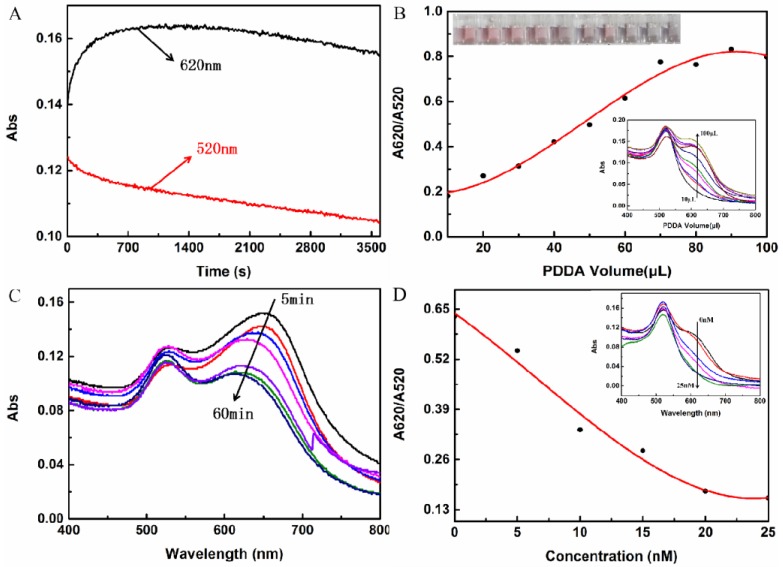
Effect of (**A**) incubation time after the addition of AuNPs, (**B**) PDDA dosage, (**C**) aptamer and PDDA incubation time, and (**D**) aptamer concentration (nM).

To explore the optimum reaction concentration, the reaction times of PDDA and AuNPs, the PDDA dose, the time of aptamer and PDDA incubation, and the aptamer concentration were optimized for 1.52 nM PDDA at 25 °C [23]. The degree of shift in the peak at 520 nm (A620/A520) and the stability of the curve were used as the criteria for evaluating the performance of these conditions. It was observed that AuNPs aggregated after the addition of PDDA solution. First, the absorption peak at 620 nm (red line) increased and then decreased, and the absorption peak decreased at 520 nm. Therefore, observations of the absorption value were performed after 5 min of incubation with AuNPs (Figure 3A).

The effect of PDDA on the color development system was studied using PDDA at a concentration of 1.52 nM, as shown in Figure 3B. As the amount of PDDA increased, the absorption peak at 620 nm increased continuously. When the amount of PDDA was 70 μL, the curve tended to the S type. It can be seen from the color change of solution that the color of the solution became obviously blue when the amount of PDDA was 80 μL. Furthermore, when the amount of PDDA was greater than 70 μL, the absorption value at 620 nm tended to be stable. Therefore, 70 μL PDDA at a concentration of 1.52 nM was subsequently selected.

The combination time of aptamer and PDDA is shown in Figure 3C. The absorption peaks at 520 and 620 nm reached a relatively stable state when the incubation time was 45 min. Therefore, 45 min was chosen as the optimal incubation time for the aptamer and PDDA.

The concentration of the aptamer was optimized at the amount of 100 μL, and the results are shown in Figure 3D. When the aptamer concentration was 20 nM, it could completely bind to PDDA in the solution. Because excessive amounts of the aptamer in the solution can affect the competitive binding of PDDA to Cd(II), the aptamer concentration of 20 nM was considered to be the optimal concentration for binding to PDDA.

### 2.4. Colorimetric Smartphone Detection of Cd(II)

**Figure 4:**
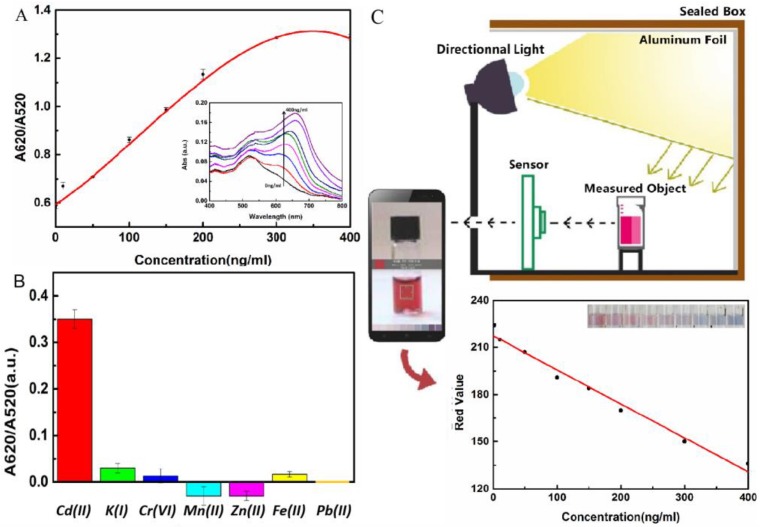
The standard curve and selectivity of the aptamer/nanogold-based colorimetric assay for detection of Cd(II). (**A**) The standard curve with different concentrations of Cd(II) using the proposed colorimetric method with UV−vis spectra. (**B**) Selectivity of the colorimetric detection of Cd(II). (**C**) Schematic illustration and the standard curve with different concentrations of Cd(II) using the proposed colorimetric method combined with a smartphone.

The color of the solution reflecting the degree of aggregation of the AuNPs was concentration-dependent on Cd(II). As shown in Figure 4A, the A620/A520 value increased when the concentration of Cd(II) increased. When the Cd(II) concentration increased to 300 ng/mL, the signal gradually reached a stable level, and the calibration curve tended to be S-shaped with relative standard deviations lower than 3.0% (*n* = 3). With the smartphone readout method, the R value was quantified using the mobile phone ColorAssist app to analyze the solution with the discoloration of Cd(II) in the RGB mode. Because of the substantial influence of light, a black box was designed with a fixed light source. The fixed light source was arranged in the box to fix the position of colorimetric ware. The sample collection hole was reserved for color collection by the mobile phone. The RGB value of the photo was read by the color recognition app on the mobile phone. The R value related to the concentration of Cd(II) in the solution. The Cd(II) concentration was plotted on the abscissa, and the R value was plotted on the ordinate, as shown in Figure 4C. The method had a good linear correlation in the range of Cd(II) concentrations of 1–400 ng/mL, and the standard curve equation was *y* = 217.462 − 0.2217*x* (r^2^ = 0.9794). Therefore, combined with a smartphone, equipment that was portable, economical, and visual was developed for the qualitative detection of concentrations based on colorimetric Cd(II) solutions.

To evaluate the selectivity of the proposed method, the influence of different interfering ions on the detection of Cd(II) was studied (Figure 4B). Under the optimized conditions, other competitive metal salts, including 100 ng/mL KNO_3_, K_2_Cr_2_O_7_, Mn(NO_3_)_2_, Zn(NO_3_)_2_, Fe(NO_3_)_2_, and Pb(NO_3_)_2_, were used. It was found that the method caused low, significant peak shifts (<10%) for K(I), Cr(VI), Mn(II), Zn(II), Fe(II), and Pb(II). This result indicated that the method had good selectivity. 

### 2.5. Sample Analysis

Rice and water samples were used to evaluate potential application of the aptamer in the detection of actual samples. The positive samples were obtained from the Testing and Evaluation Center of China Inspection and Quarantine Research Institute and also analysed according to the National Safety Standard of China (GB5009.15-2014 and GB/T 5750.6-2006). As shown in Table S1, the method based on the aptamer and gold nanoparticles for the detection of Cd(II) was consistent with the results of the National Safety Standard.

## 3. Discussion

Aptamer-based colorimetric assays provide novel opportunities for the detection of various target analytes at trace levels by utilizing AuNPs as signal transducers [24]. At same time, colorimetric methods can easily be imaged using portable smartphone or digital cameras because they can easily be designed to suit integrated smartphone applications [25]. It has been demonstrated that digital image analysis using a digital camera or a smartphone could allow for quantitative analysis by reading out the color information, especially the red, green, and blue (RGB) value of the detection zone. The RGB values provided information on the analyte’s concentration based on the RGB channel or greyscale value; this gave the concentration of the analyte based on the corresponding greyscale values [26,27,28]. Aggregation of the AuNPs in this assay is crucial for the determination of trace amounts of Cd(II). Detection depends on the full and stable complementary hybridization between the aptamer, PDDA, Cd(II), and AuNPs. In this work, PDDA is a kind of cationic polyelectrolyte that can cause aggregation of AuNPs. Without Cd(II), the aptamer can hybridize with PDDA by electrostatic interaction to form a “duplex” structure, and the AuNPs added subsequently do not aggregate. When Cd(II) was present in the assay solution, due to the formation of the Cd(II)–aptamer complex, the presence of free PDDA in the solution initiated the aggregation of AuNPs, changing its color from wine red to blue. Within a certain concentration range, the concentration of Cd(II) in the solution changed with the color of the solution along a concentration gradient. Photos were captured by the smartphone ColorAssist app software, and the R value of the RGB mode was used to realize a rapid, quantitative determination of Cd(II). Aggregation of gold nanoparticles was further verified by TEM characterization and dynamic light-scattering (DLS) particle size analysis. When Cd(II) preferentially bound to the aptamer to form a stem-loop structure, and PDDA was released from the aptamer–PDDA complex and bound to the AuNPs, which caused aggregation of the AuNPs, the color of the AuNP solution gradually became blue. The RGB values of the AuNP solution changed with the color of the AuNP solution. Synthetic nucleic acid aptamers represent a unique class of high-affinity ligands. They have demonstrated potential in applications ranging from therapy, targeted drug delivery, and sensors to diagnostic reagents [29]. Aptamer performance is directly related to the specificity of the method. This work used the Cd(II) aptamer, which was screened by Systematic Evolution of Ligands by Exponential Enrichment(SELEX) technology [7]. CD measurement results indicated that Cd(II) could bind to the aptamer, which interfered with bases from the transition of strong π–π* with ribodesose, thus resulting in the decrease of CD peak. The specificity of aptamer against other competitive metal ions was carried out. With the proposed method, the aptamer showed good selectivity to K(I), Cr(VI), Fe(II), Mn(II), Zn(II), and Pb(II). Some key parameters affecting the detection performance of the aptamer-based method were also optimized prior to the final setting up of the detection method, for example, the reaction times of PDDA and AuNPs, the PDDA dose, the time of aptamer and PDDA incubation, and the aptamer concentration. Under optimal conditions, combined with the smartphone readout, the R values were dependent on the concentration of Cd(II), showing a linear range from 1–400 ng/mL of Cd(II) with a linear equation of *y* = 217.462 − 0.2217*x* (r^2^ = 0.9794). Moreover, this approach was applied for the detection of Cd(II) in rice and water samples. Rice is one of the main fundamental foods of human beings and is one of the most important cereals. Recently, an extensive survey of Cd(II) content in domestic rice samples was performed by Zou et al. [30]. In addition, due to rapid industrialization, Cd(II) is generally widespread and commonly found in groundwater [31]. The experimental results demonstrated that digital image analysis using a smartphone could allow for quantitative analysis by reading out the color information, especially the RGB value.

## 4. Materials and Methods 

### 4.1. Materials

The 5′-CTCAGGACGACGGGTTCACAGTCCGTTGTC-3′ aptamer was obtained from Shenggong Bioengineering Technology (Shanghai, China). Chloroauric acid and sodium citrate were purchased from Strem Chemicals, Inc. The polydiene dimethyl ammonium chloride (PDDA) solution was purchased from Macklin Biochemical Co., Ltd. (Shanghai, China). The 0.22 μm polypropylene (PP) Syringe Filter was obtained from Agela Technologies (Tianjin, China). Drinking water quality control and rice quality control samples were purchased from the Chinese Academy of Inspection and Quarantine Test Evaluation Center. The cadmium standard solution was from the National Nonferrous Metals and Electronic Materials Analysis and Testing Center. Ultrapure water was obtained from Milli-Q Element (Millipore, Boston, MA, USA). All chemicals were of analytical grade or higher. 

### 4.2. Characterization

Transmission electron microscope (TEM) images were recorded on a Tecnai G2 F20(FEI). Dynamic light scattering patterns were made on a laser light-scattering instrument (American Brookhaven Instruments Corporation). The circular dichroism graph was produced on a circular dichroism instrument (JASCO J-715, Tokyo, Japan). UV-vis spectra were measured on a UV-vis spectrophotometer (UV2550, Shimadzu, Kyoto, Japan). Cd(II) was detected on an atomic absorption spectrometer (AA-6800, Shimadzu), according to the National Safety Standard of China (GB5009.15-2014 and GB/T 5750.6-2006), and using a smartphone with the proposed method.

### 4.3. Synthesis of AuNPs

AuNPs were prepared according to the reported literature procedure with few modifications [32,33]. Typically, an aqueous solution of gold chloric acid (0.01%, 100 mL) was added to a 250 mL round-bottom flask. Then, the solution was heated to 100 °C, and a sodium citrate solution (1% wt, 4 mL) was added while stirring. The reaction mixture was boiled for 40 min, then cooled and filtered through a 0.22 μm polypropylene membrane. The deposit was collected by centrifugation (10,000 rpm, 20 min) and stored at 4 °C.

### 4.4. Sample Preparation for Cd(II)

The water samples were directly measured according to the colorimetric smartphone method and the National Safety Standard of China (GB/T 5750.6-2006). The rice samples were weighed and digested according to the National Safety Standard of China (GB5009.15-2014). In total, 0.5 g of rice was immersed in a mixture of 20 mL of nitric acid and 6 mL of perchloric acid in a 100 mL beaker. Then, the sample solution was put in a graphite furnace with an internal temperature of 250 °C. The reaction continued until 0.5 mL of a transparent, slightly yellow solution was obtained. Then, the formed solution was cooled to room temperature for analysis by the atomic absorption spectrometer and colorimetric smartphone method.

### 4.5. Colorimetric Smartphone Analysis

One hundred microliters of aptamer solution (20 nM) was mixed with 100 µL sample solution in a plastic tube. Then, this solution was diluted to 330 µL with a Tris-HCl buffer (pH 7.4) and incubated at 25 °C for 30 min. Subsequently, 70 µL PDDA solution (1.52 nM) was added and incubated at 25 °C for 45 min. Finally, 100 µL AuNPs stock solution was added. After incubation at 25 °C for 5 min, the absorption spectra in the range from 400 to 800 nm and the absorbance kinetics for 520 and 620 nm peaks were measured with a UV-vis spectrophotometer. The color change of the sample was scanned and analyzed by a smartphone equipped with a ColorAssist app. The R value (RGB color model) of each specific color was instantaneously obtained.

## 5. Conclusions

In this work, a highly selective and sensitive colorimetry method was developed for measuring Cd(II) based on the aptamer combined with colorimetry measured on a smartphone. Recently, smartphones have been used in the development of portable apparatuses for absorption detection and microscopic imaging [25]. As a selective molecular recognition element, aptamer has become an efficient alternative of traditional antibody in various methods such as electrochemical biosensors [34], surface plasmon resonance biosensor [35], colorimetric [36], chemiluminescence [37], etc. To the best of our knowledge, few previous attempts have been performed to determine analyte based on the aptamer combined with colorimetry-smartphone. The results here obtained had further demonstrated its potential for practical application in visual or quantitative detections with the aptamer and smartphone. Furthermore, this proposed approach is remarkably cheaper than traditional instruments. This method may have wide applicability in colorimetry detection. 

## Supplementary Materials

The following are available online, Figure S1: (A) secondary structure of Cd(II) aptamer predicted using Mfold software. The part marked with red line has folded into a stem-loop structure. (B) CD spectra of (a) 10 μM aptamer solutions, (b) 10 μM aptamer and 50 μg/mL Cd(II) solution, (c) 50 μg/mL Cd(II) solution., Table S1: Detection of Cd(II) in rice and drinking water.
