# Supplementary material for: Highly Selective, Aptamer-Based, Ultrasensitive Nanogold Colorimetric Smartphone Readout for Detection of Cd(II)
Source: Molecules. 2019 Jul 29;24(15):2745. doi: 10.3390/molecules24152745 (PMC6695641; doi:10.3390/molecules24152745)
Supplement: Supplementary file 1 [file molecules-24-02745-s001.pdf]

Electronic Supplementary Information

Highly Selective Aptamer Based Ultrasensitive Nanogold Colorimetry-Smartphone Readout for Detection of Cd (II)

Lu Xu[1,2], Jun Liang [2], YongHui Wang[1], ShuYue Ren[1], Jin Wu[1], HuanYing Zhou[1,*], and Zhixian Gao[1*]

1 Tianjin Key Laboratory of Risk Assessment and Control Technology for Environment and Food Safety, Tianjin Institute of Environmental & Operational Medicine, Tianjin, 300050, China

2 State Key Laboratory of Food Nutrition and Safety, Tianjin University of Science and Technology, Tianjin, 300222, China

* Correspondence: zhouhytj@163.com; gaozhx@163.com.; Tel.: +86-022-84655191

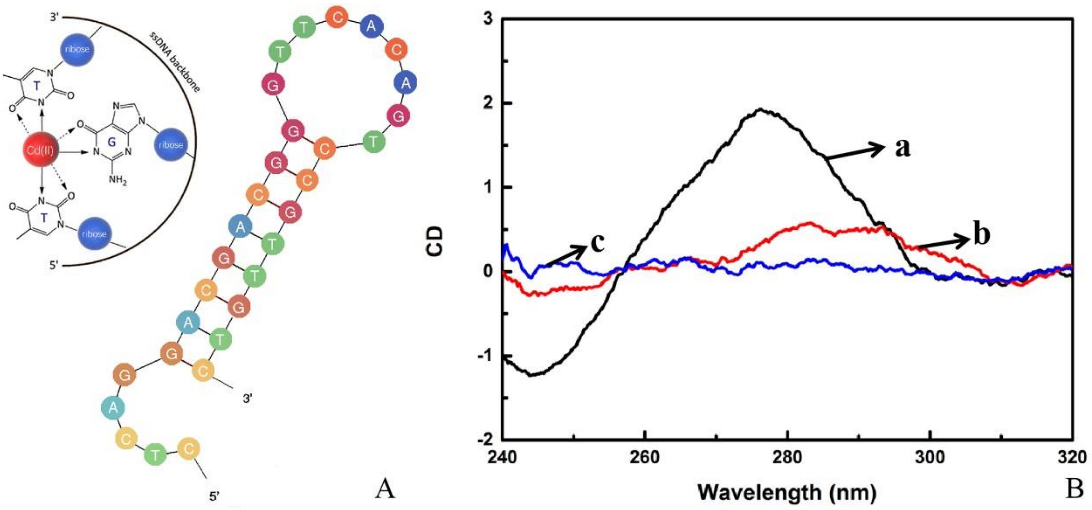

Figure S1 (A) Secondary structure of Cd (II) aptamer predicted using Mfold software. The part marked with red line had folded into a stem-loop structure. (B) CD spectra of (a) 10 μM aptamer solutions, (b) 10 μM aptamer and 50μg/mL Cd (II) solution, (c) 50μg/mL Cd (II) solution.

Table S1. Detection of Cd (II) in rice and drinking water

Method	Rice(mg/kg)	Water(ng/mL)
Nation Standard method	0.488	15.969
This method	0.455	15.200
